# Unique Cellular and Biochemical Features of Human Mitochondrial Peroxiredoxin 3 Establish the Molecular Basis for Its Specific Reaction with Thiostrepton

**DOI:** 10.3390/antiox10020150

**Published:** 2021-01-20

**Authors:** Kimberly J. Nelson, Terri Messier, Stephanie Milczarek, Alexis Saaman, Stacie Beuschel, Uma Gandhi, Nicholas Heintz, Terrence L. Smalley, W. Todd Lowther, Brian Cunniff

**Affiliations:** 1 Center for Structural Biology, Department of Biochemistry, Wake Forest School of Medicine, Medical Center Blvd., Winston-Salem, NC 27157, USA; kinelson@wakehealth.edu (K.J.N.); ugandhi@wakehealth.edu (U.G.); tsmalley@wakehealth.edu (T.L.S.J.); 2 Department of Pathology and Laboratory Medicine, University of Vermont Cancer Center, Larner College of Medicine, University of Vermont, 149 Beaumont Ave., Burlington, VT 05405, USA; Terri.Messier@med.uvm.edu (T.M.); stephanie.milczarek@med.uvm.edu (S.M.); alexis.saaman@gmail.com (A.S.); grnmtntarheel@gmail.com (S.B.); nicholas.heintz@med.uvm.edu (N.H.); 3 Wake Forest Baptist Comprehensive Cancer Center, Medical Center Blvd., Winston-Salem, NC 27157, USA

**Keywords:** mitochondrial reactive oxygen species, peroxiredoxin 3, pro-oxidant therapy, thiostrepton

## Abstract

A central hallmark of tumorigenesis is metabolic alterations that increase mitochondrial reactive oxygen species (mROS). In response, cancer cells upregulate their antioxidant capacity and redox-responsive signaling pathways. A promising chemotherapeutic approach is to increase ROS to levels incompatible with tumor cell survival. Mitochondrial peroxiredoxin 3 (PRX3) plays a significant role in detoxifying hydrogen peroxide (H_2_O_2_). PRX3 is a molecular target of thiostrepton (TS), a natural product and FDA-approved antibiotic. TS inactivates PRX3 by covalently adducting its two catalytic cysteine residues and crosslinking the homodimer. Using cellular models of malignant mesothelioma, we show here that PRX3 expression and mROS levels in cells correlate with sensitivity to TS and that TS reacts selectively with PRX3 relative to other PRX isoforms. Using recombinant PRXs 1–5, we demonstrate that TS preferentially reacts with a reduced thiolate in the PRX3 dimer at mitochondrial pH. We also show that partially oxidized PRX3 fully dissociates to dimers, while partially oxidized PRX1 and PRX2 remain largely decameric. The ability of TS to react with engineered dimers of PRX1 and PRX2 at mitochondrial pH, but inefficiently with wild-type decameric protein at cytoplasmic pH, supports a novel mechanism of action and explains the specificity of TS for PRX3. Thus, the unique structure and propensity of PRX3 to form dimers contribute to its increased sensitivity to TS-mediated inactivation, making PRX3 a promising target for prooxidant cancer therapy.

## 1. Introduction

Thiostrepton (TS) is an FDA-approved, thiazole antibiotic produced by Streptomycetes. A recent study reported by Corsello et al. tested the ability of 4518 drugs from the Drug Repurposing Hub at the Broad Institute to kill 578 cancer cells lines [1]. TS showed significant efficacy in 403 tumor cells lines. Thus, TS was highlighted as “a drug of greatest interest for mechanistic follow-up.” Importantly, previous work from our groups identified peroxiredoxin 3 (PRX3) as a key molecular target of TS [2,3]. Our data demonstrated that TS irreversibly crosslinks PRX3 through its active site Cys residues to form inactive covalent dimers, leading to increased mROS levels, reduced FOXM1 expression and anti-tumor activity in both in vitro and in vivo malignant mesothelioma (MM) tumor models.

**Figure 1:**
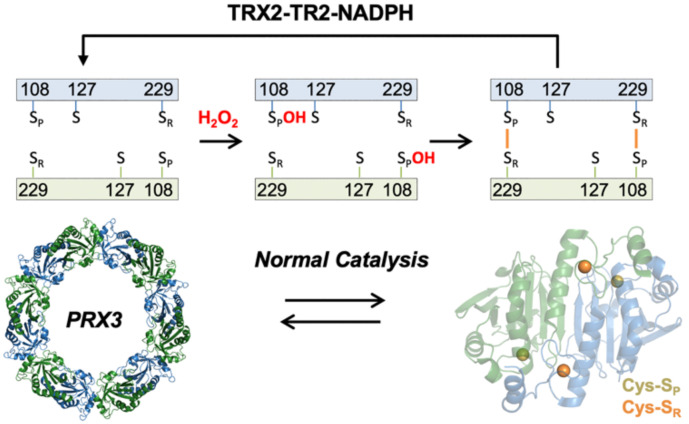
Catalytic cycle of PRX3. Hydrogen peroxide reacts with the peroxidatic Cys residue (Cys108-S_P_) to form a Cys-sulfenic acid intermediate (Cys108-S_P_OH). The resolving Cys residue (Cys229-S_R_) then reacts to form a disulfide. Disulfide bond formation facilitates collapse of the dodecamer to form 6 dimers; reduction of the disulfide by the thioredoxin 2-thioredoxin reductase 2-NADPH (TRX2-TR2-NADPH) reducing system shifts the equilibrium back toward dodecamer. Note that each active site can form a disulfide bond in the absence of TS. In the presence of TS, the data herein supports the formation of an asymmetric dimer with 1 disulfide and 1 TS-mediated crosslink.

PRX3 like all other PRXs share a common first step in catalysis in which the peroxidatic cysteine residue attacks a molecule of hydroperoxide to form a Cys sulfenic acid intermediate (Cys-S_P_OH) (Figure 1) [4]. This intermediate then reacts with the resolving Cys residue (Cys-S_R_). For the PRXs 1–4 (called the typical 2-Cys or PRX1 class), the disulfide bond formed is intermolecular across the subunit interface of the head-to-tail homodimer. Reduction of the disulfide-bonded dimers is catalyzed by thioredoxin 1 (TRX1) in the cytosol and thioredoxin 2 (TRX2) in the mitochondria [5].

Despite the mechanistic and primary sequence similarity, the 2-Cys mechanistic class members differ in: (i) their oligomeric state (e.g., PRX3 is active as a dodecamer, while PRX2 is a decamer), (ii) the thioredoxin-thioredoxin reductase (TRX-TR) system used, (iii) their specificity for hydroperoxide substrates, (iv) disulfide bond formation and reduction rates, (v) susceptibility to hyperoxidation, and (vi) subcellular distribution [4,6,7,8,9]. PRX1 and PRX2 reside in the cytoplasm. PRX3 is exclusively expressed in the mitochondrial matrix, and PRX4 is expressed in the endoplasmic reticulum. In contrast, PRX5 contains both mitochondrial and peroxisomal targeting sequences and belongs to a different class of PRXs, where the resolving Cys residue is in the same subunit and an intramolecular disulfide bond is formed during catalysis [4,10]. Because of the high degree of similarity between the PRX active sites and the different roles for these proteins in the cell, the identification of selective PRX inhibitors is both highly challenging and critical to the development of PRX-based therapeutics.

In this study, we demonstrated that TS cytotoxicity is correlated with expression levels of PRX3 and mitochondrial ROS (mROS) levels using a panel of normal mesothelial and patient-derived malignant mesothelioma (MM) cell lines. This activity is not dependent upon the mitochondrial membrane potential. Western blot analysis of PRXs 1–5 shows that only the formation of the PRX3-TS adduct is potentiated by gentian violet (GV), but this reaction does depend upon the mitochondrial membrane potential. Detailed biochemical studies with purified PRXs 1–5 support the observed cellular specificity. In particular, the higher pH of the mitochondrial compartment and the unique oligomeric state dynamics and redox properties of PRX3 make it the most sensitive to TS adduction. Since PRX3 is overexpressed in most cancers, targeting PRX3 with a specific inhibitor represents a significant therapeutic opportunity.

## 2. Materials and Methods

### 2.1. Reagents and Chemicals

Thiostrepton (Millipore, Burlington, MA, USA), Gentian Violet (Sigma, St. Louis, MO, USA), 2-[2-[4-(trifluoromethoxy)phenyl]hydrazinylidene]-propanedinitrile (FCCP, Sigma), S-Methyl Methanethiosulfonate (MMTS, TCI Chemicals, Portland, OR, USA), Bovine Serum Albumin (BSA, GOLDBIO, St. Louis, MO, USA), TCEP (Fisher, Waltham, MA, USA), DTT (Fisher) Optima HPLC grade water (Fisher), hydrogen peroxide (Honeywell, Charlotte, NC, USA), NADPH (Roche, Basel, Switzerland), IPTG (Fisher), glucose 6-phosphate dehydrogenase (Alfa Aesar, Haverhill, MA, USA), glucose 6-phosphate (ACROS Organics, Morris Plains, NJ, USA), BioGel P6 Resin (BioRad, Hercules, CA, USA).

### 2.2. Cells and Cell Culture

Primary and immortalized mesothelial cells and malignant mesothelioma cells were cultured as previously described [2,3]. The STR profiles are of human origin, analysis was performed in the Vermont Integrative Genomics Resource DNA Facility and was supported by University of Vermont Cancer Center, Lake Champlain Cancer Research Organization, and the UVM Larner College of Medicine. The STR profiles matched previously annotated DNA fingerprints [3].

### 2.3. Immunoblotting

Cells were plated in 6 well plates at a density of 200,000 cells per well. After 24 h, cells were treated with indicated compounds as described in the text. Cell lysates were harvested at indicated time points using RIPA buffer (50 mM Tris-HCl, 150 mM NaCl, 1 mM EDTA, 1% NP-40, 0.25% Sodium deoxycholate, 0.1% sodium dodecyl sulfate, in deionized (DI) water) for reducing samples to be analyzed by reducing SDS-PAGE. For samples to be analyzed by non-reducing SDS-PAGE, cells were incubated in 100 mM S-Methyl Methanethiosulfonate (MMTS, TCI), to prevent artifactual oxidation of reduced cysteines, diluted in warm PBS for 20 min followed by lysis with RIPA buffer containing 100 mM MMTS. Protein concentrations were determined via Bradford Assay (ThermoScientific, Rockford, IL, USA). Lysates (15 µg protein/well) were resolved by SDS-PAGE under reducing conditions (Dithiothreitol (DTT) was omitted for non-reducing gels) on 4–12% gradient Bis-Tris Midi gel (Invitrogen, Carlsbad, CA, USA) at constant 200 V for 50 m. The gel was transferred to a PVDF membrane at constant 1A for 50 min, blocked with 5% BSA diluted in 1× Tris-buffered saline with 1% Tween-20 (TBS-T) for a minimum of one h, and incubated with appropriate primary antibody (Table S1) at indicated dilution in 5% BSA TBS-T at 4 °C overnight. The membrane was washed with 1× TBS-T for 1 h, incubated with appropriate secondary antibody at indicated dilution for 1 h, and washed again with 1× TBS-T for 1 h. Membranes were incubated with ECL Reagent (ThermoScientific, Rockford, IL, USA) and visualized using a GE Amersham Imager chemiluminescent detection system. Blots were washed with 1× TBS-T and re-probed with loading control antibody to verify equal protein loading. Densitometry was performed using ImageJ (NIH).

### 2.4. Cell Viability Assays

Cell Lines were plated in 96-well plates (Corning, Kennebunk, ME, USA) at a density of 2500 cells per well. The following day, cells were treated with test compounds diluted in complete media followed by incubation for 48 h. Post-incubation cells were washed with PBS (Corning Cellgro, Manassas, VA, USA), fixed with 3.0% formaldehyde (Fisher BioReagents, Fair Lawn, NJ, USA) in PBS, and stained for 30 min with 0.1% crystal violet (Acros Organics, Fair Lawn, NJ, USA) in water. Crystal violet stain was removed, and plates were washed with H_2_O and allowed to dry. To quantify cell viability, plates were imaged using the Lionheart Plate reader (BioTek Instruments, Winooski, VT, USA) and/or analyzed by absorbance at 540 nm (crystal violet dye dissolved in 100% methanol) using the Synergy HTX plate reader (BioTek Instruments, Winooski, VT, USA). To determine the effective cytotoxic concentration (EC_50_) of test compounds the data were plotted using a 4-parameter non-linear regression model using GraphPad Prism7 software (GraphPad Software, San Diego, CA, USA).

### 2.5. Agilent Seahorse XF Cell Mito Stress Test Assay

A total of 6000 cells of each cell line were plated in 80 μL complete media into individual wells of a XF96 cell culture microplate excluding cells from 4 corner control wells (5 technical replicates per assay). The following day, cells were treated with test compounds diluted in complete media followed by incubation for indicated time. Post-incubation cells were washed 2× with warm, sterile filtered Seahorse XF RPMI media supplemented with 1 mM pyruvate, 2 mM glutamine, and 10 mM glucose at pH 7.4. 80 μL of supplemented Seahorse XF RPMI assay media was added back to each well. Cells were allowed to equilibrate for 1 h in a 37 °C CO_2_ free humidified incubator before loading into a XFe96 extracellular flux analyzer temperature adjusted to 37 °C (Seahorse Bioscience, Billerica, MA, USA). Sensor cartridges were equilibrated with XF calibrant for 24 h before loading with inhibitors. Inhibitor concentrations were titrated to determine optimal drug concentrations to establish bioenergetic profiles (data not shown), final well concentrations used were 1.0 μM oligomycin, 1.0 μM carbonyl cyanide-4 (trifluoromethoxy) phenylhydrazone (FCCP), and 0.5 μM rotenone and antimycin A. Oxygen and proton concentrations were measured every 8.5 min for 1 h and 35 min, inhibitors (oligomycin, FCCP, rotenone/antimycin A, respectively) to measure mitochondrial bioenergetics were added to the plates through the microinjection ports at the indicated time points. Oxygen consumption rates (OCR) and spare reserve capacity (difference between maximal and basal OCR) are shown. Cells were imaged using the Lionheart FX Automated Microscope (BioTek Instruments, Winooski, VT, USA) to confirm cell adherence before and after assay.

### 2.6. MitoSOX Red Detection of Mitochondrial ROS

Indicated cell lines were loaded with 5 µM MitoSOX Red (Invitrogen, Carlsbad, CA, USA), diluted in DMSO, in tissue culture medium for 30 min. Cells were washed with Hanks buffered salt solution with calcium and magnesium (HBSS) and incubated with 1% BSA in HBSS. MitoSOX Red fluorescence was collected at 593 nm following excitation with the RFP filter cube (ex 531-nm) using the Lionheart Plate reader (BioTek Instruments, Winooski, VT, USA). Cell number was counted and used to normalize MitoSOX Red signal to cell number. Primary mesothelial cells immediately lifted from the plate when incubated with MitoSOX Red and therefore were not amenable to this procedure. 

### 2.7. Expression and Purification of Recombinant Proteins

Wild-type human PRX1, PRX1 C83E, and wild-type PRX2 were expressed without a tag from the pET17b vector and purified as previously described [2]. Untagged wild-type PRX3 (residues 62–256) was expressed in C41 (DE3) cells from the PTYB21 expression vector with an N-terminal Intein fusion and purified as previously described [9]. The engineered dimer of PRX3 with and without the resolving Cys (PRX3-S139E/A142E and PRX3-S139E/A142E/C229S) as well as the engineered dimer of PRX2 (PRX2-T82E) were purified from BL21 Gold (DE3) *Escherichia coli* cells with a non-cleavable N-terminal His-tag from the pET15b vector, as previously described [2]. *E. coli* thioredoxin 2 (Trx2, the trxC gene product) was expressed and purified as previously described [11]. For all purifications, HisPur Cobalt affinity resin (Thermo Scientific, Waltham, MA, USA) was used. PRX fractions were pooled, concentrated, flash-frozen with liquid nitrogen, and stored at −80 °C until use. All proteins were stored in a buffer without dithiothreitol (DTT) except for the PRX3 C229S variant. Wild-type PRX3 was oxidized to disulfide by 1.5 equivalents H_2_O_2_ prior to storage.

*E. coli* thioredoxin reductase (eTR) was expressed from the pET15pp vector [12]. BL21 Gold (DE3) cells containing the TrxR construct were grown at 37 °C in 2 12 L fermenters until the OD_600_ = 0.8 and induced with 0.5 mM isopropyl 1-thio-β-D-galactopyranoside at 18 °C for 18–20 h. The cells were lysed in 100 ml of Affinity buffer (20 mM HEPES, pH 7.9, 500 mM KCl) supplemented with 1–2 mg FAD, 10% glycerol, 0.1% Triton X-100, 0.1 mM PMSF and 0.1 mM benzamidine using an Emulsiflex C5 homogenizer (Avestin, Inc, Ottawa, ON, Canada). After centrifugation, proteins were purified from the supernatant by affinity chromatography using HisPur cobalt affinity resin (Thermo Scientific). The N-terminal His tag was cleaved by adding 1 mg HRV-3C protease for every 15 mg of protein and allowing the cleavage to proceed overnight at 4 °C during dialysis into buffer containing 20 mM HEPES, 100 mM NaCl, pH 7.5. The fractions containing the protein of interest were further purified by Q-Sepharose FF and Superdex 200 columns (both GE Healthcare). The final storage buffer was 25 mM Hepes pH 7.5, 100 mM NaCl.

The human PRDX4 gene (residues 84–271) was codon optimized for expression in *E. coli* by GenScript and subcloned into the pET15pp vector. The resultant protein contained a non-cleavable, N-terminal His-tag. The engineered PRX4 dimer (T155E) was generated by Genscript from the same vector. His-tagged PRX4 and PRX4-T155E were purified as described above for eTR except that the buffers were not supplemented with FAD and no protease was added. The mature form of recombinant human PRX5 protein containing a non-cleavable N-terminal 6X His-tag was expressed and purified as previously described [13].

### 2.8. In Vitro Turnover Assays with TS and PRX

Reactions comparing wild-type PRXs 1–5 and engineered PRX dimers contained 100 µM PRX, 5 µM *E. coli* TRX2, 0.5 µM *E. coli* TR, and a NADPH regenerating system composed of 3.2 mM glucose 6-phosphate, 3.2 U/ml glucose 6-phosphate dehydrogenase and 0.4 mM NADPH. Samples were incubated at 37 °C with either 0.25 mM TS or an equivalent volume of DMSO (<1%). To determine the pH dependence of TS, the engineered dimers of PRX1s 1–3 were diluted to 100 µM with 5 mM TCEP in BPCDN buffer at various pH values. BPCDN buffer contains 20 mM phosphate (pK_a2_ = 7.2), 20 mM boric acid (pK_a_ = 9.0), 20 mM sodium citrate (pK_a2_ = 5.2), 0.2 mM diethylenetriamine pentaacetate (DTPA), and 200 mM sodium chloride and allows for a single buffer solution to be used across pH values ranging from 4.2–10 [14]. Assay components were pulsed with successive additions of 50 µM H_2_O_2_ to induce turnover of PRX3 and incubated at 37 °C for 10–15 min between H_2_O_2_ additions. Reactions were stopped by the addition of 5× SDS loading buffer containing 100 mM DTT, heated to 100 °C for 10 min, and proteins were separated by 12% SDS-PAGE and stained for total protein using GelCode Blue (Life Technologies, Carlsbad, CA, USA). The intensity of the non-reducible PRX dimer was divided by the summed intensity of all PRX bands within each lane. Results are presented as mean values ± SD from a minimum of three independent replicates. Data were analyzed by two-way ANOVA with a Tukey HSD post-hoc correction using GraphPad Prism version 7.04. 

### 2.9. Reaction of TS with PRX3-SH vs. PRX3-SOH

The PRX3 EE C229S variant was reduced for 10–20 min at ambient temperature with 20 mM DTT. The sample was then passed through a Bio-Gel P6 spin column equilibrated in 50 mM Tris, pH 8.0 to remove excess DTT. To prevent spontaneous oxidation of the reduced protein, all buffers for this experiment were made using HPLC-grade water. To test the reduced sample, PRX3-EE-C229S was diluted to a final concentration of 20 µM and incubated for 90 min at 37 °C with either 200 µM TS, 20 mM dimedone, or an equivalent amount of DMSO. To test the sulfenic acid form of the protein, 20 µM reduced PRX3-EE-C229S was treated with 30 µM H2O2 for 3 min prior to the addition of DMSO, dimedone, or TS. Samples were stored at −20 °C overnight, and 10 µL of each sampler was passed through a 100 µL BioGel P6 column equilibrated in 25 mM ammonium acetate in HPLC water. Samples were analyzed on a Bruker Autoflex MALDI-TOF mass spectrometer in positive ion and linear acquisition mode using a matrix containing 20 mg/mL sinapic acid, 50% acetonitrile, and 0.1% formic acid in water. 

### 2.10. SEC-MALS Analysis to Measure PRX Size Distribution

Approximate molecular weights of PRX proteins were determined using size exclusion chromatography coupled with multiangle light scattering (SEC-MALS) analysis. SEC-MALS buffers were made in HPLC water and contained 50 mM HEPES, pH 7.5 and 100 mM NaCl. PRXs were diluted into SEC-MALS buffer prior to resolution on a TSK Gel 4000 SW gel filtration column (8 μm particle size, 7.8 × 30 cm, Tosoh Biosciences, Tokyo, Japan). Chromatographic separation was performed over 35 min at a flow rate of 0.5 mL/min, monitoring elution spectrophotometrically at A_280_ and MALS using a HELIOS II detector (Wyatt Technology). Molecular weights were estimated for prominent peaks (determined from the A_280_ traces) from MALS data using Astra 6 software (Wyatt Technology, Goletta, CA, USA).

To measure the oligomeric state of reduced and oxidized PRXs at pH 7,0, 7.5, or 8.0 PRX proteins were diluted to 2 mg/mL (90–93 µM) in SEC-MALS buffer to a final volume of 0.12 mL at the indicated pH values. Reduced samples included 10 mM DTT and oxidized samples included 1.2 equivalents of H_2_O_2_. Chromatographic separation was performed on 100 µL (0.2 µg) PRX protein in the absence of reducing agents.

To measure the oligomeric state of PRX with sub-stoichiometric amounts of disulfide formation (Figure S6), wild-type, untagged PRX3 or PRX2 were reduced with 20 mM DTT for 10–20 min at RT. Samples were then passed through a 1 mL BioGel P6 spin column to remove excess DTT and to exchange into SEC-MALS buffer pH 7.4 (PRX2) or pH 8.0 (PRX3). PRXs were diluted to 100 µM in 120 µL SEC-MALS buffer and 24 µL of a 5 × stock of peroxide was added to obtain the desired final concentration of H_2_O_2_. PRX2 (100 µM) was titrated with final peroxide concentrations of: 0 (reduced), 20 µM (2/10 equivalents), 40 µM (4/10 equivalents), 60 µM (6/10 equivalents), 80 µM (8/10 equivalents), or 100 µM (1 equivalence) H_2_O_2_. PRX3 (93 µM) was titrated with final peroxide concentrations of: 0 (reduced), 15.5 µM (2/12 equivalents), 31 µM (4/12 equivalents), 46.0 µM (6/12 equivalents), 62 µM (8/12 equivalents), 77.5 µM (10/12 equivalents), or 93 µM (1 equivalence) H_2_O_2_.

## 3. Results

### 3.1. PRX3 Expression and Mitochondrial ROS Levels Correlate with Sensitivity to Thiostrepton

**Figure 2:**
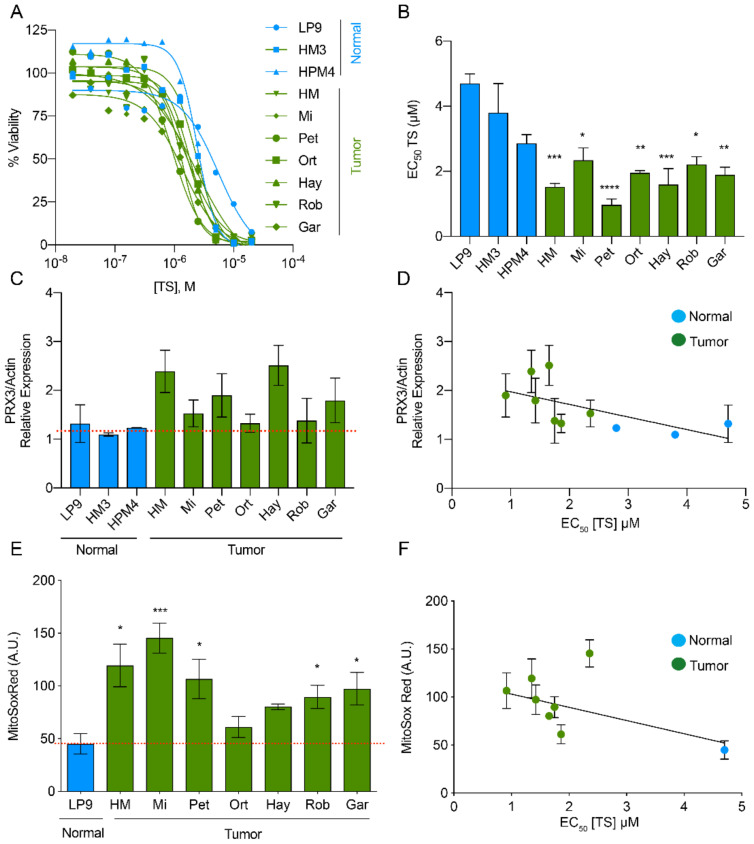
Mitochondrial ROS levels and PRX3 expression correlates with sensitivity to thiostrepton. (**A**) Dose-response curves of normal and MM tumor cell lines to thiostrepton (TS). (**B**) EC_50_ values for TS in normal and MM cell lines (* *p* < 0.05, ** *p* < 0.01, *** *p* < 0.001, **** *p* < 0.0001, *n* = 4 biological replicates). (**C**) Relative PRX3 protein expression levels in MM tumor and normal cells (normalized to actin; See Figure S1 for representative Western blot). Red dotted line for reference to mean expression of PRX3 in normal cell lines (n = 2 replicates, ±SEM). (**D**) Relationship between PRX3 expression and EC_50_ values for TS in normal and MM tumor cells (*p* = 0.025, *n* = 2). (**E**) MitoSOX Red levels in normal and MM tumor cell lines (* *p* < 0.05, *** *p* < 0.001, *n* = 4). Red dotted line for reference to mean MitoSOX Red levels in normal cells (Of note, primary mesothelial cells were not amenable to analysis using MitoSOX Red). (**F**) Relationship between MitoSOX Red levels and EC_50_ values for TS in normal and MM tumor cells (* *p* = 0.028, *n* = 4).

Aggressive tumors rely on increased antioxidant expression for survival under otherwise inhospitable redox conditions [15,16]. To determine if there was differential sensitivity to TS, we profiled a panel of primary mesothelial, normal immortalized mesothelial and MM cell lines using cell-viability assays. The concentration of TS required to kill 50, 90 and 99% (EC_50_, EC_90_, EC_99_) of cells was calculated from dose-response curves (Figure 2A,B and Figure S1A). Primary and immortalized non-tumorigenic mesothelial cells were moderately sensitive to TS with EC_50_ values between ~2.8–4.7 µM. Tumorigenic MM cell lines, representative of both pleural and peritoneal disease from sarcomatoid and biphasic histologic subtypes, were more sensitive to TS compared to normal cell lines, with EC_50_ values of ~0.9–2.4 µM TS. EC_99_ values extrapolated from survival curves show MM cell lines are on average ~10 × more sensitive to TS than normal mesothelial cells at their respective EC_99_ concentrations (Figure S1A).

We next examined PRX3 protein expression levels in normal and MM cell lines by immunoblotting (Figure S1B). PRX3 protein expression is elevated in most MM cell lines compared to normal mesothelial cells (Figure 2C). We compared TS sensitivity (EC_50_) to PRX3 protein expression levels and identified a linear relationship between the two variables (Figure 2D). The slope of the line comparing TS sensitivity and PRX3 expression was significantly non-zero (*p* = 0.025). The levels of mROS in normal and MM cell lines were also determined by staining their mitochondria with MitoSOX Red, a redox-sensitive fluorescent dye (most reactive with superoxide). The fluorescence intensity of this dye was also significantly increased in the majority of MM cell lines as compared to LP9 normal mesothelial cells (Figure 2E), indicating higher mROS levels in MM tumor cell lines. We compared TS sensitivity (EC_50_) to MitoSOX Red fluorescence intensity and identified a linear relationship between the two variables (Figure 2F). The slope of the line comparing the levels of mROS and sensitivity to TS was significantly non-zero (*p* = 0.028). In addition, mitochondrial bioenergetics in normal and MM cell lines was evaluated using Seahorse Extracellular Flux analysis (Figure S1C). The spare reserve capacity of mitochondria in MM cells was significantly lower when compared to normal mesothelial cells (Figure S1D). Interestingly, this readout of the ability to respond to bioenergetic stress did not significantly correlate with TS sensitivity. Altogether, these data provide evidence that elevated PRX3 protein expression and increased mitochondrial oxidant levels in MM cell lines correlate with sensitivity to TS. 

### 3.2. In Vitro (Cellular) Specificity of TS for Mitochondrial PRX3

**Figure 3:**
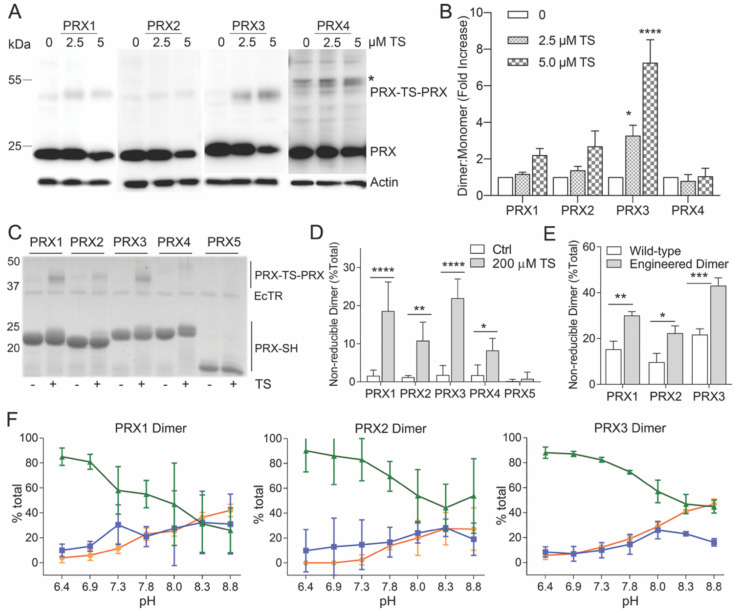
Specificity of TS for cellular and recombinant mitochondrial PRX3. (**A**) Western blots of PRXs 1–4 from MM tumor cells (HM cell line) incubated with indicated concentrations of TS for 24 h. Lysates were run on reducing SDS-PAGE gels (* = PRX-TS-PRX indicated the TS-mediated, cross-linked dimer, * = non-specific band in PRX4 sample). (**B**) Quantification of PRXs 1–4 covalent crosslink by TS in MM tumor cells (*n* = 3). Significance is shown relative to no TS control for each PRX. (**C**) Human PRXs 1–5 (100 µM) were cycled at 37 °C at pH 8 in the presence or absence of 50 µM TS, 5 µM *E. coli* TRX2, 0.5 µM *E. coli* TR, a NADPH regenerating system, and 6 successive additions of 50 µM H_2_O_2_ and run on a reducing SDS-PAGE gel. PRX4 was purified with an N-terminal His tag. PRXs 1–3 & 5 were untagged. (**D**) Quantification of the TS crosslink (dimer:total PRX ratio) for wild-type PRXs 1–5 (mean values ± SD, *n* = 3. (**E**) Quantification of TS crosslink for wild-type proteins and their dimeric variants (mean values ± SD, n = 3). WT and engineered dimers of PRXs 1–3 were untagged. (**F**) pH-dependence of TS adduct formation for the untagged, engineered dimers of PRX1, PRX2, and PRX3. The amount of reduced protein monomer (green), TS monomer adduct (blue) and TS crosslinked dimer (orange) were determined by SDS-PAGE in triplicate, see Methods for details. * *p*-value < 0.05, ** *p*-value < 0.005, *** *p*-value < 0.0005, and **** *p*-value < 0.0001.

TS induces the formation of an irreversible PRX3-TS-PRX3 protein species, in vitro and in vivo, that migrates at ~45 kDa on reducing SDS-PAGE gels, the expected molecular weight of PRX3 dimers (Figure 3A) [2]. The TS-mediated PRX crosslink persists despite the treatment of samples with 10 mM DTT in SDS buffer prior to separation by SDS-PAGE electrophoresis, indicating that the covalent TS complex is highly stable once formed. To determine whether TS was able to crosslink other typical 2-Cys PRXs in cells, MM cells were treated with 2.5 or 5 µM TS for 24 h and cell lysates were collected. We immunoblotted the cell lysates with antibodies specific for cytosolic PRX1 and PRX2, mitochondrial PRX3 and the endoplasmic reticulum (ER) localized isoform PRX4. TS induced the formation of a dose-dependent, non-reducible PRX3 dimer in MM cells (Figure 3A,B). In contrast, TS induced non-significant modifications to cytosolic PRXs 1 and 2 and had no observable effect on PRX4. These data provide evidence that TS preferentially reacts with mitochondrial PRX3 and has minimal reactivity with cytosolic or ER resident PRXs in MM cells. 

### 3.3. Specificity of TS for Recombinant PRX3

Although TS exhibits a clear preference for PRX3 in cells, it is not known whether this specificity arises from innate differences in the structure and reactivity of PRX3 compared to other PRX proteins. We tested the ability of TS to form crosslinks between recombinant human PRXs 1–5. Importantly, PRXs 1–3 did not contain any extra residues or affinity tags, since the inclusion of tags can influence the oligomerization and kinetics of typical 2-Cys PRXs [17,18]. Because changes in pH are known to perturb the equilibrium between dimer and decamer/dodecamer, the protonation state of the peroxidatic and resolving Cys residues, and the activity of the E. coli TRX/TR system, our initial assays were all performed at pH 8.0 [2]. Multiple cycles of enzymatic turnover were accomplished by the addition of NADPH and six subsequent additions of 50 µM hydrogen peroxide (See Methods for details). The formation of a TS-induced crosslink was visualized by the appearance of a non-reducible dimer on a reducing SDS-PAGE gel (Figure 3C). PRXs 1–3 all showed the appearance of some cross-linked dimer (12–23%, Figure 3D) in the presence of TS. In contrast, no significant TS crosslink was observed for PRX4. PRX5 belongs to a different class of PRXs but is of interest because it does reside in the mitochondria [10]. Because the disulfide in human PRX5 is intramolecular, a TS crosslink would be expected to increase the size of the monomer rather than induce the formation of a non-reducible dimer. No TS adduct was observed for PRX5 by either gel electrophoresis or mass spectrometry (Figure 3C and Figure S2). It is important to reiterate here that these experiments were performed at pH 8.0, which mimics the mitochondrial environment. Thus, the PRX isoforms (PRXs 1, 2 and 4) that normally function in a lower pH environment may behave differently when tested under conditions that mimic their normal environment, as demonstrated in the experiments below.

### 3.4. Thiostrepton Preferentially Adducts Dimeric PRX Species

In solution, PRXs 1–4 proteins exist in an equilibrium between dimers and higher order oligomers that include decamers (PRXs 1, 2, and 4) and dodecamers (PRX3). This equilibrium is influenced by numerous conditions including protein concentration, ionic strength, post-translational modifications, redox state and pH [4]. In particular, the dodecamer of oxidized PRX3 is less stable and dissociates into dimers [6]. Therefore, we tested whether PRX1 and PRX2 would become more reactive to TS, if present in the dimeric form. Previous published results from our groups and others have shown that the introduction of one or two negatively charged residues into the dimer-dimer interface disrupts decamer/dodecamer formation [19,20,21,22]. The resulting PRX1 C83E, PRX2 T82E, and PRX3 S139E/A142E variants are exclusively dimeric independent of oxidation state and under the conditions used in the studies herein (Figure S3). At pH 8.0, all the engineered dimer variants formed TS-crosslinks more efficiently than the wild-type proteins (Figure 3E). 

### 3.5. TS Adducts PRX Dimers Only at Mitochondrial pH

Performing in vitro experiments at pH 8 is only relevant for mitochondrial PRX3, where pH values range between 7.5 and 8.2; the pH of the cytosol is significantly lower (7.0–7.2) [23]. To determine whether the pH differences between the subcellular compartments contribute to the observed selectivity of TS in vivo, TS reactivity with human PRXs 1–3 were evaluated across a range of pH values (Figure S4). For these experiments, we used TCEP as an alternative reducing system because it is able to efficiently reduce disulfides across a broad pH range [24], unlike the TRX-TR system [25]. TS was more reactive with WT PRXs 1–3 at the higher pH values (5–15% PRX-TS-PRX adduct) found in the mitochondria compared to the lower pH values found in the cytosol (2–5% PRX-TS-PRX adduct, Figure S4). To decouple the pH dependence in reactivity with TS from the pH dependence of the dimer-oligomer equilibrium between the proteins, we evaluated the TS reactivity with the engineered dimers of PRXs 1–3 across the same range of pH values (Figure 3F). The dimeric versions of PRX1, PRX2, and PRX3 were all more reactive at higher pH values (20–50% PRX-TS-PRX adduct at pH 8 vs. 0–7% at pH 7). Thus, the data support that the pH differences between the mitochondria and the cytosol are likely to contribute to the intracellular specificity of TS for PRX3. We have also shown that the dimeric form of PRXs is the species that most readily reacts with TS. 

### 3.6. TS Reacts with the Reduced Cys Thiolate in PRX3, but Not the Sulfenic Acid Intermediate

**Figure 4:**
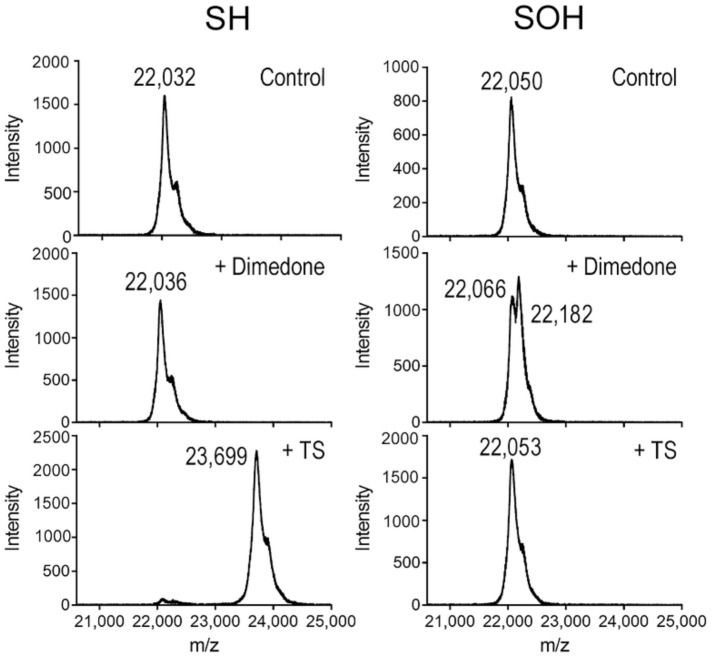
TS reacts with reduced Cys thiolate in PRX3, but not the sulfenic acid intermediate. MALDI-TOF MS analysis of the reaction of TS with untagged engineered dimer of PRX3 C229S in different oxidation states. Pre-reduced, untagged PRX3 engineered dimer lacking the Cys-S_R_ residue was oxidized with 1.5 equivalents of H_2_O_2_ to form a stable sulfenic acid intermediate. PRX3 with a thiol (SH) or sulfenic acid (SOH) were split and incubated with 1% DMSO (control), 20 mM dimedone, or 0.2 mM thiostrepton. Dimedone was added to confirm that sulfenic acid was present only in the samples treated with H_2_O_2_.

We have reported that TS adduction by PRX3 requires both the peroxidatic (Cys108) and resolving (Cys229) cysteines, and that the crosslinked adduct accumulates over time with multiple rounds of enzymatic turnover [2]. Both the reduced cysteine thiolate and the cysteine sulfenic acid intermediate in PRX proteins (Figure 1) are nucleophilic and form a covalent complex with electrophilic thiol blocking reagents, including *N*-ethyl maleimide [26]. In this set of experiments, we asked if the reaction of PRX3 with TS requires the Cys108 to be in the reduced form (Cys-SH) or as the Cys-sulfenic acid intermediate (Cys-SOH). To generate a stable Cys-sulfenic acid species, the PRX3 engineered dimer with the resolving Cys229 residue mutated to Ser was pre-reduced and reacted with 1.5 equivalents of H_2_O_2_. TS was then incubated with either reduced protein or the stabilized sulfenic acid intermediate at pH 8.0 at 37 °C for 45 min and unreacted TS was removed using a Bio-Gel P-6 column. The mass of the proteins was determined by MALDI-TOF mass spectrometry (Figure 4). Because MALDI-TOF is a relatively low-resolution instrument and the sulfenic acid intermediate is not stable during ionization, the presence of sulfenic acid was confirmed by the addition of dimedone, a small molecule that reacts with sulfenic acid but not thiol/thiolate [27]. Only the peroxide-oxidized samples showed an increase in mass consistent with the addition of one dimedone (143 Da vs. theoretical of 140 Da), indicating the presence of a stable sulfenic acid. An increase in the mass consistent with the addition of one TS molecule (1660 Da versus theoretical of 1664 Da) only occurred when PRX3 was in the reduced state, consistent with the reduced thiol but not the sulfenic acid acting as the nucleophile to attack TS.

### 3.7. PRX3 Is More Likely to Be Found as Dimers than PRX1 or PRX2

**Figure 5:**
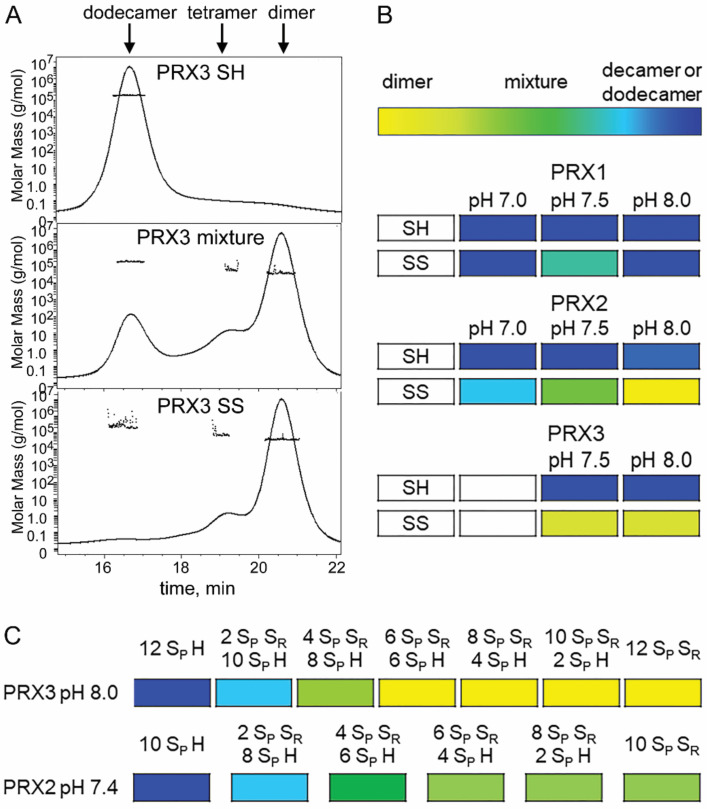
PRX3 is more likely to be dimer than PRX1 or PRX2. (**A**) SEC-MALS analysis of the influence of oxidation state on the distribution of oligomeric states for untagged wild-type PRX3 at pH 8.0. Shown is the elution profile OD_280_ trace for reduced (top), partially oxidized (middle panel) and oxidized (bottom panel). The dodecameric species elutes at ~17 min, while the dimer elutes at ~21 min. The mass for each species is indicated by the dots (left, y-axis). (**B**) Comparison of oligomeric states for PRX1, PRX2 and PRX3 as a function of pH and oxidation state (SH versus SS). The oligomeric state at each concentration ranged from fully dimeric (yellow) to fully (do)decameric (dark blue), with many samples exhibiting a mixture of the two species. The color gradient represents the approximate relative values of dimer and decamer within the mixtures. (Elution profiles for all samples are shown in Figure S3). All proteins were untagged. (**C**) Titration of PRX3 and PRX2 with peroxide at their typical cellular compartment values. Pre-reduced PRX2 and PRX3 were treated with increasing equivalents of peroxide. Relative amounts of dimer and (do)decamer in each sample are represented by the same color gradient as (**B**). The elution profiles for each sample are shown in Figure S5. PRX3 was not analyzed at pH 7.0 because of instability and precipitation at this pH.

The preference of TS for reduced and dimeric PRX is particularly interesting, as fully reduced PRXs 1–4 are predominantly found as decamers/dodecamers [4]. Based on our data we propose that PRX3 is more frequently found as a reduced dimer than PRX1 or PRX2 due to either differences in the oligomeric structure and/or differences in subcellular pH. In order to test this hypothesis, we used size-exclusion chromatography with multi-angle light scattering (SEC-MALS) with different pH buffers. For example, using this technique, one can separate the PRX3 dodecamer (~17 min) from the dimer (~21 min) and measure the mass of each species (Figure 5A). The oligomeric states of reduced and oxidized PRX1 and PRX2 were determined at pH 7.0, 7.5 and 8.0 (Figure 5B and Figure S3). PRX3 was tested only at pH 7.5 and 8.0 since it was not stable at lower pH values in our hands. Reduced PRXs 1–3 were fully decameric/dodecameric at all pH values tested. In contrast, the oligomeric state of the oxidized PRX proteins differed greatly as a function of pH. Oxidized PRX1 was predominantly decameric at all pH values. Oxidized PRX2 was predominantly decameric at pH 7.0, and the amount of dimer observed increased with pH, with dimer predominating at pH 8.0. In contrast, oxidized PRX3 was fully dimeric at both pH 7.5 and 8.0. We next tested the reactivity of the PRXs 1–3 engineered dimers across the pH range using the same SEC-MALS assay (Figure S3). All engineered dimers were dimeric at all pH values tested. 

Taken together, these data support that for the oxidized wild-type proteins at the physiological relevant pH, the equilibrium for PRX3 is shifted more towards dimer than PRX1 and PRX2. The preceding experiments utilized either fully reduced or fully oxidized proteins, a state most likely not observed in cells when PRXs are constantly undergoing active turnover. It is also important to note, however, that some number of reduced active sites are necessary to react with TS (Figure 1). Therefore, TS would be most reactive when partial oxidation of PRX3 leads to dodecamer destabilization, while allowing a portion of the active sites to remain reduced. To test the hypothesis that the dodecameric structure of PRX3 is much less stable than the decameric structure of PRX2, the oligomeric status of PRX2 and PRX3 were measured by SEC-MALS analysis following partial oxidation at their respective cellular compartment pH. The pre-reduced proteins were treated with increasing substoichiometric amounts of H_2_O_2_, assuming that the H_2_O_2_ concentration will result in disulfide formation in an equal number of PRX active sites (Figure 5C and Figure S5). PRX1 was not measured since this isoform showed very little dimer formation even when fully oxidized (Figure 5B). At pH 8.0, PRX3 was dodecameric when fully reduced and a significant amount of dimer starts to appear upon the addition of 1/6 equivalents of H_2_O_2_ (enough to oxidize 2 of 12 active sites). PRX3 was fully dimeric after oxidation of 6 of the 12 active sites. In contrast, PRX2 still maintained a significant amount of decamer even when fully oxidized. This data and the higher pH of the mitochondria compared to the cytosol suggest that a much higher fraction of PRX3 will be dimeric than PRX2, which will be more frequently dimeric than PRX1. Therefore, PRX3 will have significantly more dimers with reduced active sites available to react with TS than other typical 2-Cys PRXs. 

### 3.8. Gentian Violet Potentiates TS Adduction of PRX3 but Not PRX1 and PRX2

**Figure 6:**
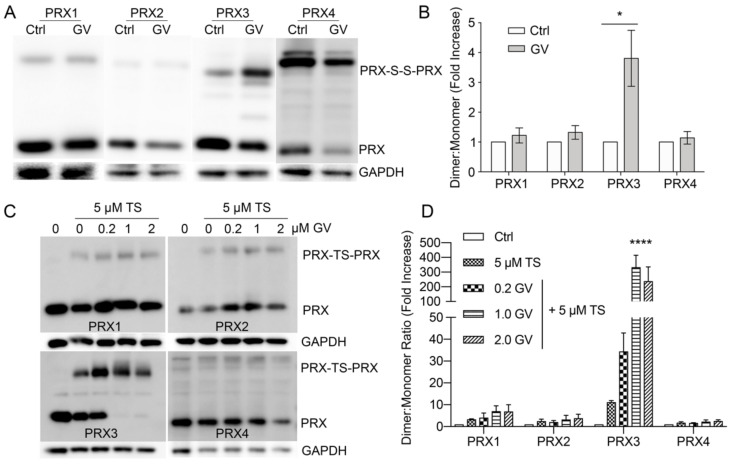
Specificity for Gentian violet (GV) to potentiate TS crosslinking of mitochondrial PRX3. (**A**) Western blots of PRXs 1–4 from MM tumor cells incubated with 1 µM GV for 24 h. Lysates were run on non-reducing SDS-PAGE gels. PRX-S-S-PRX represents the native intermolecular disulfide; the band above this indicated region in PRX4 is a non-specific band. (**B**) Quantification of PRXs 1–4 disulfide formation induced by GV under non-reducing conditions (*n* = 3). (**C**) Western blots of PRXs 1–4 from MM tumor cells incubated with 5 µM TS or 5 µM TS + indicated concentration of GV for 24 h. Lysates were run on reducing SDS-PAGE gels. (**D**) Quantification of PRXs 1–4 covalent crosslink by TS or TS + GV under reducing conditions. (* *p* < 0.05; **** *p* < 0.0001).

Treatment of MM tumor cells with GV leads to loss of TRX2 protein within 30 min [2]. When GV is used in combination with TS, the majority of PRX3 is irreversibly crosslinked by TS in MM cells [2,3]. To determine if GV can potentiate the formation of disulfide-bonded dimers in other PRX isoforms, we incubated MM cells with 1 µM GV and collected cell lysates after 24 h for immunoblotting analysis by non-reducing SDS-PAGE. Under these conditions PRXs migrate as 23–26 kDa reduced monomers and 46–50 kDa disulfide-bonded dimers (Figure 6A). Treatment with GV significantly increased the abundance of mitochondrial PRX3 disulfide-bonded dimers but had no observable effect of the formation of PRXs 1, 2, or 4 disulfide-bonded dimers (Figure 6A,B). To further evaluate the effects of GV, MM cells were incubated with increasing concentrations of GV in combination with a fixed concentration of TS for 24 h. These samples were then analyzed for TS induced PRX crosslinking by reducing SDS-PAGE and immunoblotting (Figure 6C,D). TS alone induced significant PRX3 crosslinking and minimal crosslinking of PRXs 1, 2 and 4, as described above. When TS was used in combination with GV, a dose dependent increase in PRX3 crosslinking was observed; at higher GV concentrations, all of the observed PRX3 formed covalent PRX3-TS-PRX3 adducts. No increase in PRXs 1, 2, or 4 crosslinking was observed with GV treatment. These data provide convincing evidence that TS and GV exert their cellular effects specifically on the mitochondrial TRX2/PRX3 antioxidant network.

### 3.9. Disruption of the Mitochondrial Membrane Potential Does Not Affect TS-Mediated Crosslinking of PRX3

**Figure 7:**
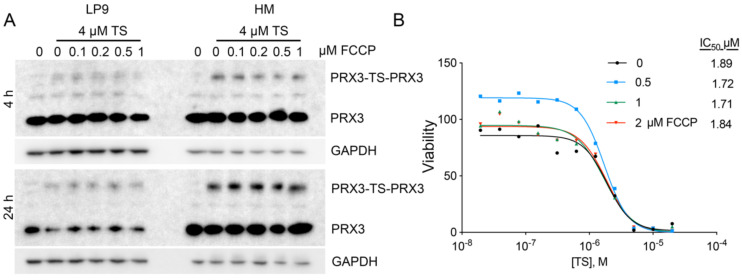
Intact mitochondrial membrane potential is not required for TS activity. (**A**) Western blots of normal LP9 and MM (HM cell line) cells incubated with 4 µM TS alone or in combination with indicated concentrations of FCCP. Lysates were collected at 4- and 24-h post incubation and separated by reducing SDS-PAGE. (**B)** Dose response curves of HM cells incubated with TS alone or in combination with indicated concentrations of FCCP. IC_50_ values (µM) are shown for indicated treatment groups.

As mitochondrial membrane potential has been shown to play a significant role in the uptake and localization of compounds to the mitochondria [28], the effects of depolarizing the mitochondrial membrane potential on TS activity were evaluated. FCCP, a well-documented proton ionophore that rapidly dissipates mitochondrial membrane potential, depolarizes mitochondrial membranes rapidly in normal mesothelial and MM cells, as assessed by Seahorse Extracellular Flux Analysis (Figure S1C,D). Crosslinking of PRX3 by TS was unaffected at both early (4 h) and late (24 h) time points in normal and MM cell lines in the presence of FCCP (Figure 7A). The cytotoxic activity of TS in the presence of multiple concentrations of FCCP was also unaffected (Figure 7B). These data indicate that TS exerts cytotoxic activity through PRX3 inhibition independent of mitochondrial membrane potential.

To investigate the contribution of mitochondrial membrane potential on the ability of GV to act on mitochondrial targets, MM cells were incubated with GV and FCCP and cell lysates were collected over time. FCCP had minor effects on GV induced PRX1 and PRX2 disulfide-bonded dimer formation (Figure S6A,B). In contrast, GV induced PRX3 disulfide bonded dimers rapidly (15 min); the level of which was sustained throughout the time course (4 h) (Figure S6C). Loss of TRX2 expression was rapid, showing reduction in TRX2 protein levels by 30 min. Co-incubation of GV with 1 µM FCCP blunted TRX2 degradation and PRX3 disulfide-bonded dimer formation throughout the entirety of the experiment. These data indicate that unlike TS, GV activity against mitochondrial targets is sensitive to mitochondrial membrane potential. This is not unexpected due to the triphenyl-structure and the positive charge of GV [29,30]. 

## 4. Discussion

The goal of this study was to determine the molecular basis for the specificity of TS for human PRX3, one key component for this candidate redox-based cancer therapy. The origins of TS specificity could come from a combination of many variables, including normal versus cancer cell ROS levels, PRX3 levels, differences in the cellular localization of TS and/or PRX enzymes and differences in the reactivity and structural features of the PRX proteins. Since the PRXs have similar reaction mechanisms (Figure 1), detailed cellular and mechanistic studies evaluating multiple PRX isoforms within cells and with recombinant proteins were necessary.

**Figure 8:**
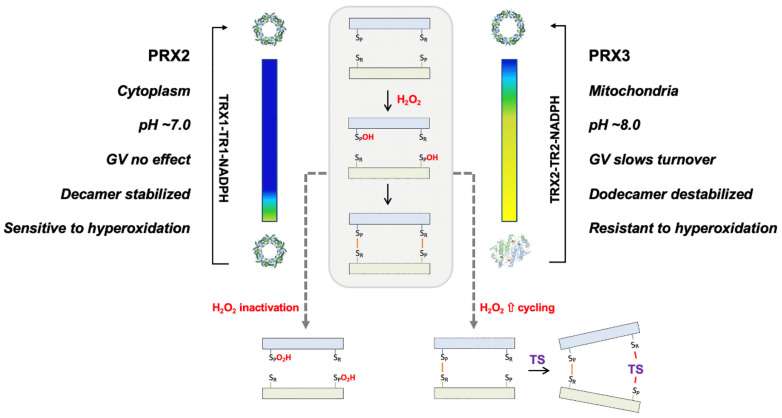
Summary of the biochemical, structural and environmental conditions that determine the specificity of TS for PRX3. The PRX3 dodecamer (blue) readily collapses to dimer (yellow; same coloring scheme used as in Figure 5), while the PRX2 decamer is more stable. In contrast to PRX2 where an increase in H_2_O_2_ cycling leads to hyperoxidation and inactivation, PRX3 can form an asymmetric dimer with one disulfide present. The other active site can then react with TS to form a covalent crosslink. Treatment with GV slows the reduction of the disulfide, further enhancing the formation of TS adducts. Double TS adducts have been observed with recombinant PRX3.

PRX3 is over-expressed in MM cells and tumors [31], and PRX3 expression levels and mROS levels correlate with TS sensitivity (Figure 2). In MM cell culture, TS preferentially adducts mitochondrial PRX3 compared to the cytosolic enzymes PRX1 and PRX2 and ER-localized PRX4 (Figure 3). Additionally, GV exerts its effects on mitochondrial TRX2, specifically increasing the abundance of partially oxidized PRX3 disulfide-bonded dimers, the preferred target of TS, without any effect on PRXs 1, 2 or 4 (Figure 8). The data provided within argue that it is possible to target mitochondrial PRX3 specifically. Moreover, tumor types with genetic and phenotypic features of increased mitochondrial oxidative stress will be more susceptible to PRX3 and/or TRX2 inhibition [16]. 

We previously showed that TS targets PRX3 and provided preliminary evidence that PRX3 dimers, versus high molecular weight dodecamers, were the preferred target of TS [2,3]. Herein, we greatly expanded the analysis to determine the specificity of TS for PRX3 versus PRX1, PRX2 and PRX4 in cells and determined the structural and biochemical features of PRX3 that modulate this specificity. TS was able to crosslink all 2-Cys isoforms (PRXs 1–4) when the reactions were conducted at the pH of the mitochondrial compartment (~pH 8.0) (Figure 3). The engineered, dimeric forms of PRXs 1–3 were all more reactive than their native counterparts, particularly at pH 8.0. When the reaction was conducted at cytosolic pH (~7.4), crosslinking of all PRX isoforms by TS was significantly attenuated. These data provide novel evidence that TS activity against PRX3 is partially driven by the elevated pH found in the mitochondrial matrix. Of interest, elevated pH levels found in tumor cells may also contribute to the therapeutic window of TS in normal versus tumor cells [32]. 

These observations led us to determine which wild-type PRX isoform can form dimers containing some reduced active sites under physiological conditions. For these studies, we used a constant concentration of 100 µM PRX, which is physiologically relevant even if somewhat low for the estimates of PRX3 mitochondrial concentrations (48–125 µM) and slightly higher than the estimated concentration of PRX1 and PRX2 in the cytosol (20–65 µM) [33,34,35]. A recent study using real-time monitoring of the PRX2 oligomerization state confirmed for the first time that PRX2 decamer-dimer oscillations occur and that oxidation favors dimers in cellulo. Of particular interest, this study confirmed that there was a measurable portion of reduced PRX2 dimers in cells, indicating that partial oxidation of PRX2 is sufficient to promote dimerization [36]. However, it should be noted that the oxidation state is only one factor and that the dimer/(do)decamer equilibrium can also be influenced by protein concentration, salt, pH and the presence of reporter and affinity tags.

Our results showing that TS reacts with the reduced thiol/thiolate in PRXs and more efficiently with the PRX dimer, led us to hypothesize that the specificity of TS is driven by structural differences in PRX3 that facilitate collapse of the dodecamer toward dimer. Previous studies have shown that the equilibrium of other oxidized typical 2-Cys PRXs is shifted toward decamer at pH 7.0 and toward dimer at pH 8.0, with this transition driven by the protonation state of a conserved His residue within the dimer-dimer interface [37]. Here we show that the pH-dependence of the dimer/(do)decamer equilibrium differs between human PRXs 1–3. While oxidized (disulfide bonded) PRX2 shows a shift from decamer at pH 7 toward dimer at pH 8, the equilibrium for oxidized PRX1 is shifted toward decamer at all pH ranges, and the equilibrium for PRX3 is shifted toward dimer (Figure 5). Importantly, only a small proportion (2/12 possible disulfides) of the PRX3 active sites are needed to react with H_2_O_2_ to facilitate collapse of the dodecamer to the constituent dimeric units. This amount of peroxide required is even lower than reported earlier by colleagues [7]. The results from the panel of human PRXs tested herein supports the proposed concept from the former study that each PRX has its own “oxidation threshold” for dissociation to the dimeric subunits [36], with PRX3 exhibiting the strongest propensity to dissociation based on our data.

Taken together, these observations (Figure 8) are also consistent with the relative insensitivity of PRX3 to hyperoxidation and inactivation when compared to PRX1, PRX2 and bacterial 2-Cys PRX homologs [6,7,8,38]. Thus, under conditions of oxidative stress, PRX1 and PRX2 are more likely to become hyperoxidized and inactivated. Moreover, hyperoxidation of PRX1 and PRX2 stabilizes the decamer, further protecting the cytosolic proteins from TS inhibition (i.e., for the subunits within the decamer that were not hyperoxidized). In contrast, our data expands upon the observation that partial oxidation of PRX3 shifts it to dimer [7], which both protects it from hyperoxidation [22] and provides a pool of reduced Cys residues available to react with TS. Decreased TRX2 expression or activity would decrease the rate of disulfide reduction further, allowing for the accumulation of partially oxidized and dimeric PRX3, as seen in our experiments using the TRX2 inhibitor GV.

Given the requirement for mitochondrial pH, we also wondered if the mitochondrial membrane potential played a role in TS and GV activity. While disruption of the membrane potential did not affect TS cytotoxicity, it did blunt the ability of GV to reduce TRX2 protein levels and potentiate the adduction of PRX3 with TS (Figure S6). While it makes sense that the loss of membrane potential blocks the uptake of the positively charged GV molecule, the mechanism by which TS enters the mitochondria is currently unknown.

In conclusion, the primary drivers for the specific reaction of PRX3 with TS in cells include its mitochondrial localization with its higher pH and the unique biochemical and structural features that protect the protein from hyperoxidation and promote the facile collapse of the dodecamer to dimer (Figure 8). Thus, these observations add novel details to the mechanism of action of TS. The reaction of PRX3 with TS can be potentiated with GV by producing more dimeric species with only one disulfide bond, suggesting that other compounds or therapies that induce oxidative stress and lower the activity of the mitochondrial TRX2-TR2 system could also synergize. Indeed, several compounds with pro-oxidant features have been shown to synergize with TS [39,40,41]. The specific features of the TS-PRX3 interaction elucidated here provide mechanistic insight into this novel drug-target interaction, provide evidence inhibitors can specifically target the PRX3 isoform and support further development of this redox-based, prooxidant therapeutic strategy for cancer [4,42]. 

## Data Availability

The data presented in this study are available on request from the corresponding authors.

## Supplementary Materials

The following are available online at https://www.mdpi.com/2076-3921/10/2/150/s1. Table S1: Sources and experimental conditions for the antibodies used in this study; Figure S1: TS sensitivity, PRX3 expression and Oxygen Consumption Rates (OCR) in MM cell lines and effect of membrane potential on TS activity; Figure S2: PRX5 does not react with TS; Figure S3: Comparison of oxidation- and pH-dependence of the oligomeric state for WT and engineered dimers of PRX1, PRX2 and PRX3; Figure S4: Comparison of the pH-dependence of TS adduct formation for WT and engineered dimers of PRXs 1–3; Figure S5: PRX3 dodecamer collapses to the dimeric form upon partial oxidation, while PRX2 decamer persists despite full oxidation; Figure S6: Effect of mitochondrial membrane potential on GV activity in MM cells (HM cell line).
