# Supplementary material for: Unique Cellular and Biochemical Features of Human Mitochondrial Peroxiredoxin 3 Establish the Molecular Basis for Its Specific Reaction with Thiostrepton
Source: Antioxidants (Basel). 2021 Jan 20;10(2):150. doi: 10.3390/antiox10020150 (PMC7909569; doi:10.3390/antiox10020150)
Supplement: Supplementary file 1 [file antioxidants-10-00150-s001.pdf]

antioxidants

MDPI

Supplementary Materials and Information

Table S1. Sources and experimental conditions for the antibodies used in this study.

Primary Antibody Name	Company	Catalog No.	Spp.	Lab Dilution	MW
Prx 1	AbFrontier	LF-MA0214	mouse-mono	1: 5,000	20-30 kDa
Prx 2	R&D Systems	AF3489	goat-poly	1: 3,000	20-30 kDa
Prx 3	AbFrontier	LF-PA0255	rabbit-poly	1: 1,000	20-30 kDa
Prx 4	Abcam	ab59542	rabbit-poly	1: 2,000	31 kDa
GAPDH	Invitrogen	MA5-15738	mouse-mono	1: 3,000	27 kDa
Actin	Invitrogen	MA5-11869	mouse-mono	1: 2,500	42 kDa

Secondary Antibody Name	Company	Catalog No.	Lab Dilution
anti-goat IgG HRP from rabbit	EMD Millipore / CalbioChem	401515	1: 3,000
anti-mouse IgG HRP from sheep	GE Healthcare	NA931	1: 3,000
anti-rabbit IgG HRP from donkey	GE Healthcare	NA934	1: 3,000

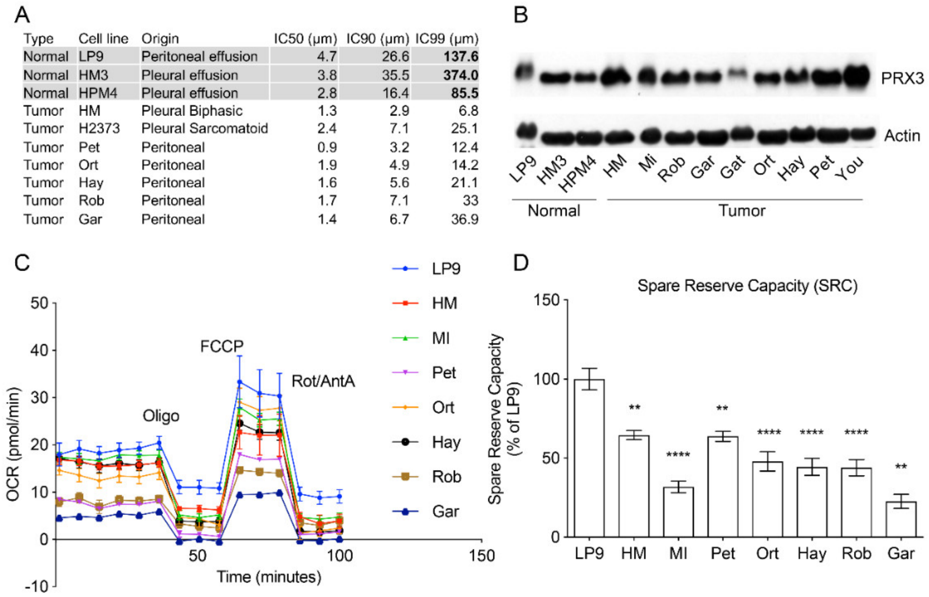

Figure S1: TS sensitivity, PRX3 expression and Oxygen Consumption Rates (OCR) in MM cell lines and effect of membrane potential on TS activity. (A) Calculated EC_{50}, EC_{90} and EC_{99} values for TS in normal and MM tumor cell lines. (B) Western blot of PRX3 and actin in normal and MM cell lines. (C) Oxygen Consumption Rates (OCR) of normal and MM cell lines collected with a Seahorse Extracellular flux Bioanalyzer. Oligomycin (Oligo), FCCP and Rotenone/Antimycin A (Rot/AntA) were added at indicated time points. (D) Spare Reserve Capacity (SRC) of normal and MM cell lines. SRC is the difference between maximum OCR and baseline OCR (n = 4 replicates). Shown as percent of control (normal LP9 cells) (** $p < 0.01$, *** $p < 0.0001$ relative to LP9 control cells, n = 4 replicates).

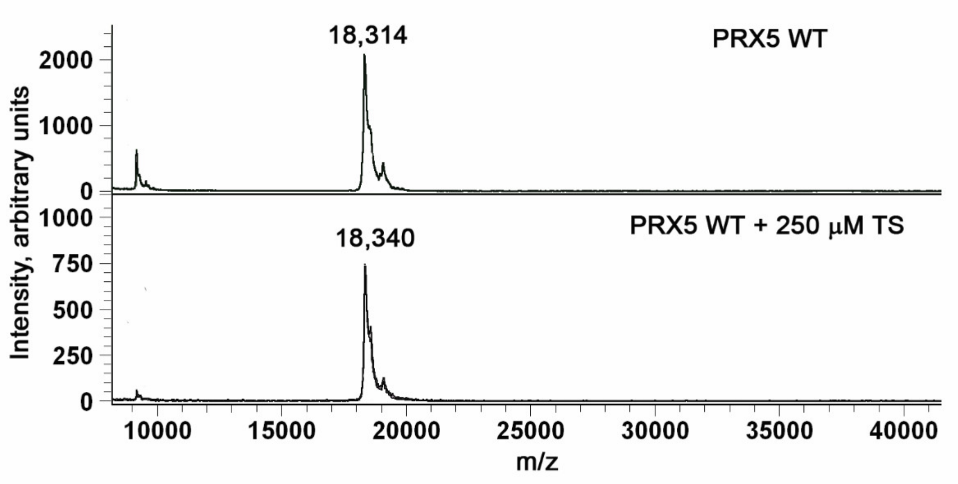

Figure S2: PRX5 does not react with TS. Wild-type recombinant PRX5 with an N-terminal 6X His tag (100 μM) was incubated at 37 °C with either 0.25 mM TS or an equivalent volume of DMSO in the presence of 5 μM *E. coli* TRX2, 0.5 μM *E. coli* TR, and a NADPH regenerating system composed of 3.2 mM glucose 6-phosphate, 3.2 U/ml glucose 6-phosphate dehydrogenase and 0.4 mM NADPH. Six successive additions of 50 μM H_2O_2 was added to induce turnover of PRX5 and incubated at 37 °C for 10-15 min between H_2O_2 additions. Samples were stored at -20 °C overnight, and 1 μL of each sample was analyzed on a Bruker Autoflex MALDI-TOF mass spectrometer in positive ion and linear acquisition modes. The mass of PRX5 (expected 18,298 Da) did not change in the presence of TS, supporting that this PRX is resistant to TS-mediated inactivation.

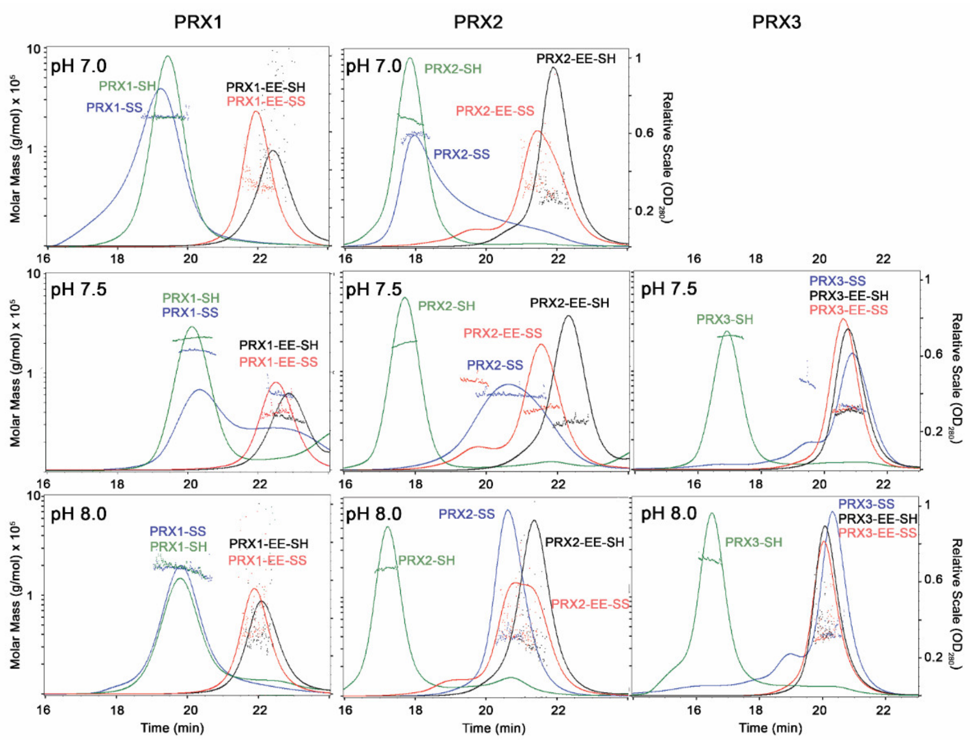

Figure S3: Comparison of oxidation- and pH-dependence of the oligomeric state for WT and engineered dimers of PRX1, PRX2 and PRX3. PRX proteins were diluted to 2 mg/mL (90-93 µM) in 20 mM HEPES, 100 mM NaCl to a final volume of 0.12 mL at the indicated pH values. Reduced samples included 10 mM DTT and oxidized samples included 1.2 equivalents of H_2O_2. SEC-MALSs analysis was performed on 100 µL (0.2 µg) PRX protein as described in Methods, using 20 mM HEPES, 100 mM NaCl at the indicated pH as the separation buffer. Shown is the elution profile for each protein monitoring spectrophotometrically at OD_{280}. The dots indicate the molar mass (left y-axis) determined. The decameric/dodecameric species elute earlier in the gradient (left peak) and the dimeric species run elute later (right peak). Molecular weights were estimated for prominent peaks (indicated by dots above each peak) from MALS data using Astra 6 software (Wyatt Technology). All engineered proteins (PRX1-C83E, PRX2-T82E, PRX3-S139E/A142E) were fully dimeric under all conditions tested.

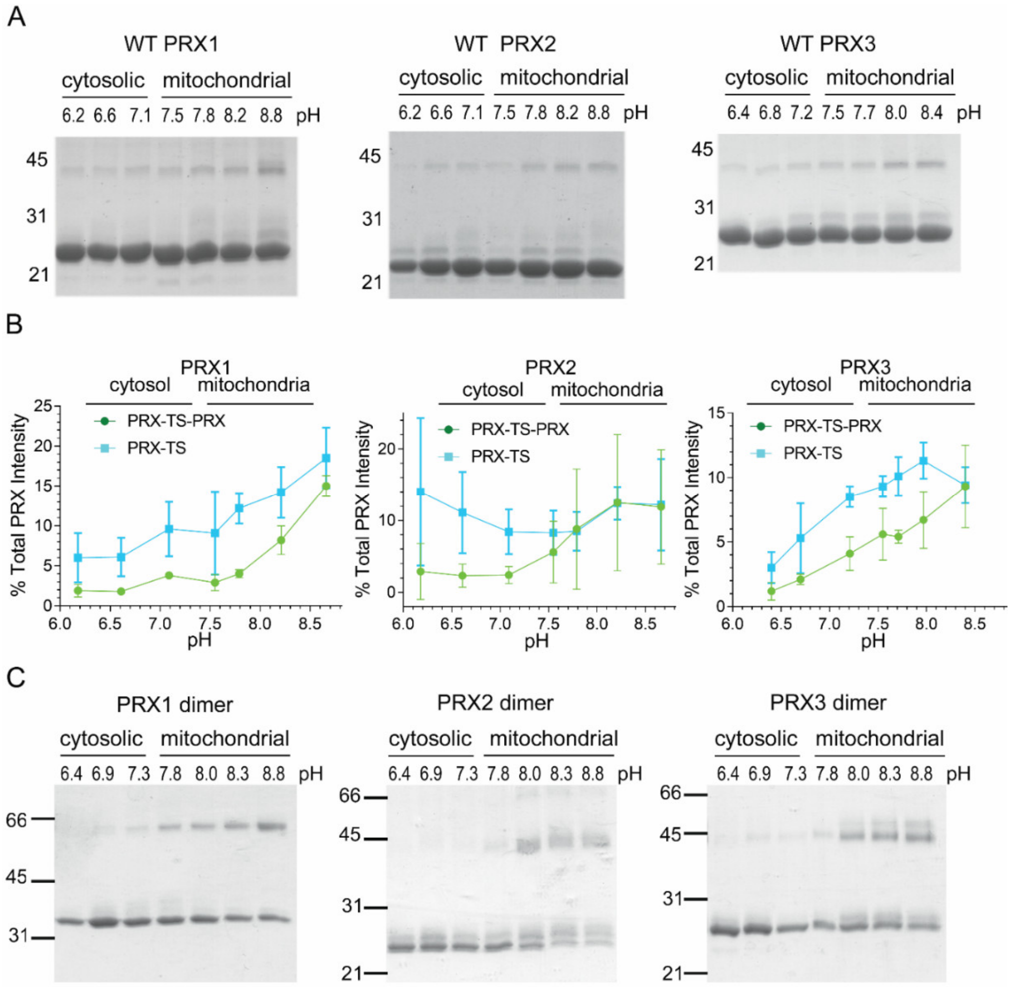

Figure S4: Comparison of the pH-dependence of TS adduct formation for WT and engineered dimers of PRXs 1-3. (A) pH-dependence of TS adduct formation for untagged, recombinant WT PRX1, PRX2, and PRX3 using TCEP as reductant. Commassie stained gels are representative of 3 independent experiments for each protein. (B) The fraction of TS monomer adduct (blue) and TS crosslinked dimer (green) for WT PRX1, PRX2 and PRX3 were quantified using ImageJ see Methods for details. Average values and standard deviation for 3 independent experiments are shown. (C). Commassie stained gels are representative of 3 independent experiments measuring the pH dependence of TS adduct formation with the engineered dimers of PRXs 1-3.

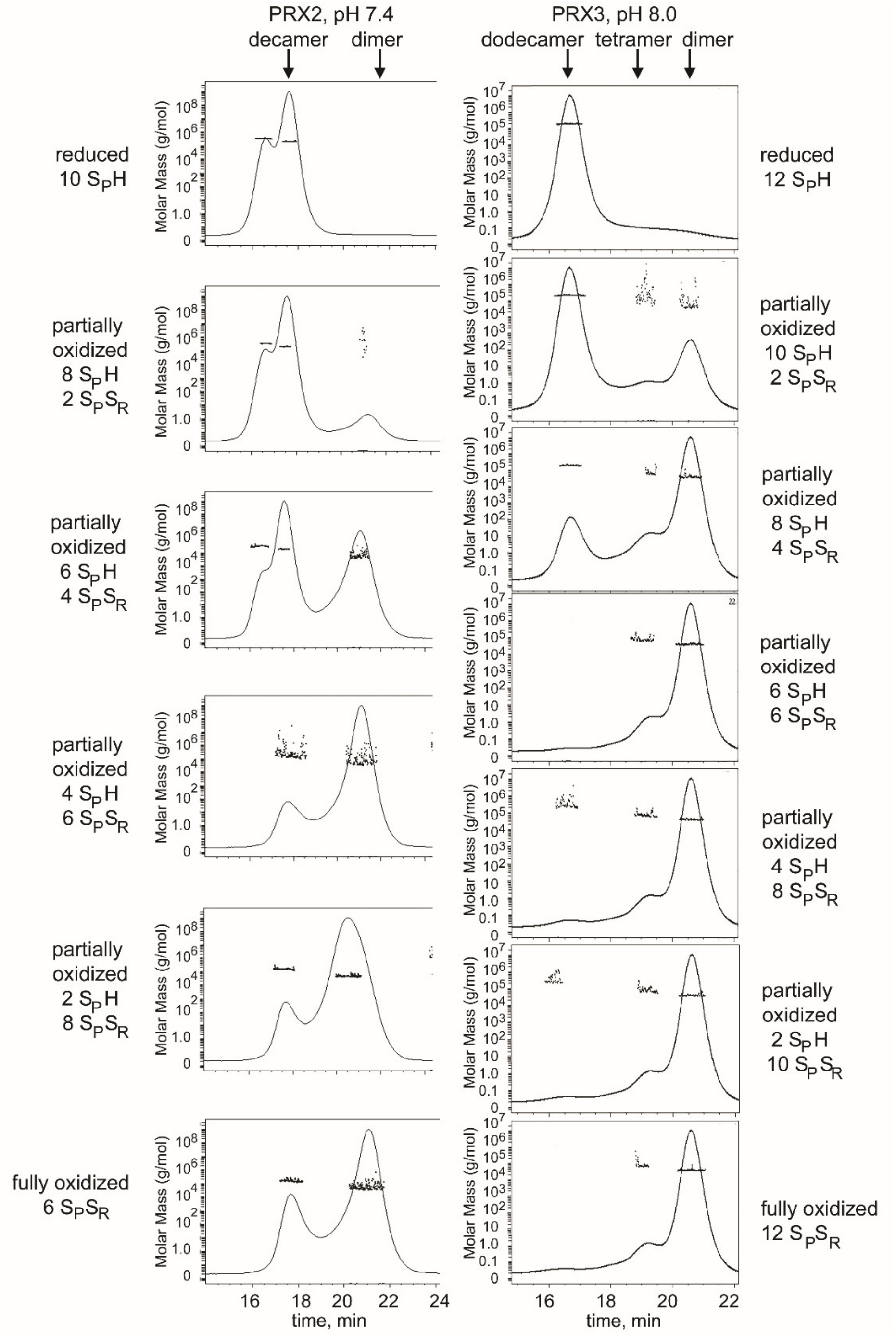

Figure S5: PRX3 dodecamer collapses to the dimeric form upon partial oxidation, while PRX2 decamer persists despite full oxidation. Either pre-reduced wild-type PRX2 or PRX3 (2 mg/ml) was incubated with sub-stoichiometric equivalents of H_2O_2 at RT for 20 min so that a fraction of the protein was oxidized to disulfide. For example, 0.4 molar equivalents of H_2O_2 was added to PRX2 with the expected result of 4 disulfides and 6 reduced thiols in the PRX2 decamer. The maximal peroxide levels added were <0.1 mM for all samples. The size distribution of the resulting samples was measured by SEC-MALS analysis (See Figure 5, Figure S3 and Methods for more details).

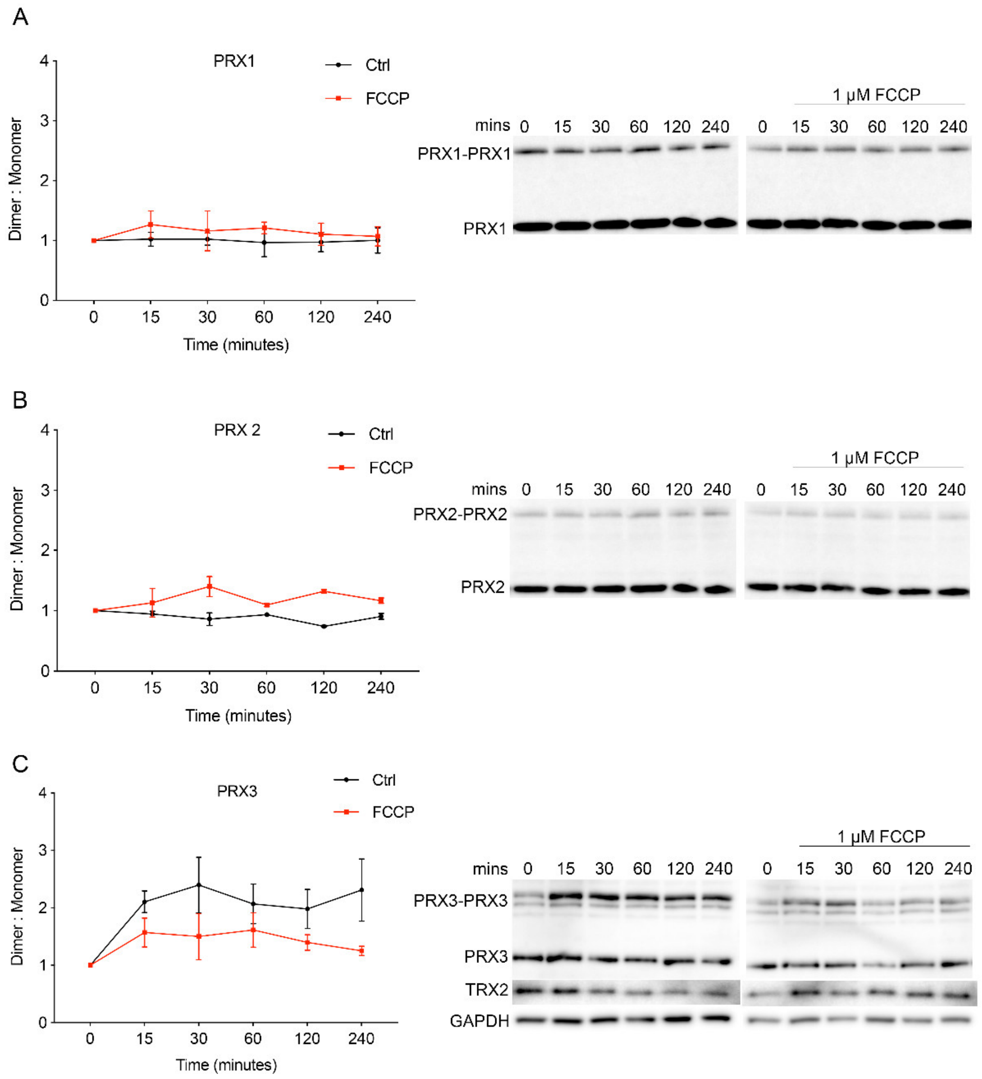

Figure S6: Effect of mitochondrial membrane potential on GV activity in MM cells (HM cell line). (A) Quantification of GV induced PRX1 disulfide-bonded dimers under control conditions (1 µM GV present) or co-incubation of 1 µM GV with 1 µM FCCP for the indicated time period (n = 3). Lysates were separated by non-reducing SDS-PAGE (right). (B) Quantification of GV induced PRX2 disulfide-bonded dimers under control conditions (1 µM GV present) or co-incubation of 1 µM GV with 1 µM FCCP for the indicated time period (n = 3). Lysates were separated by non-reducing SDS-PAGE (right). (C) Quantification of GV induced PRX3 disulfide-bonded dimers under control conditions (1 µM GV present) or co-incubation of 1 µM GV with 1 µM FCCP for the indicated time period (n = 3). Lysates were separated by non-reducing SDS-PAGE (right).
